# Interpreting functional diffusion tensor imaging

**DOI:** 10.3389/fnins.2014.00068

**Published:** 2014-04-11

**Authors:** Joonas Arttu Autio, R. Edward Roberts

**Affiliations:** ^1^Medical Research Center Oulu, Oulu University Hospital and University of OuluOulu, Finland; ^2^Department of Diagnostics, Faculty of Medicine, University of OuluOulu, Finland; ^3^Department of Diagnostic Radiology, Oulu University HospitalOulu, Finland; ^4^Division of Brain Sciences, Academic Department of Neuro-otology, Imperial College London, Charing Cross Hospital CampusLondon, UK

**Keywords:** functional diffusion tensor imaging, fractional anisotropy, BOLD, MRI, behavior

## Section

In this issue Mandl and colleagues replicated the findings of a previous study (Mandl et al., 2008) in which they explored task-related changes in fractional anisotropy (FA) along white matter (WM) tracts using functional diffusion tensor imaging (fDTI). They report increased FA in WM of thalamocortical pathways during tactile stimulation and in the optic radiations during visual stimulation, while only minor changes in mean diffusivity (MD) and blood oxygenation level dependent (BOLD) contrast were observed. Mandl and colleagues suggest that fDTI might provide a novel window on previously inaccessible WM information transfer. These findings, in addition to a number of previous reports of changes in MD with close temporal proximity to behavioral stimuli, could have a significant impact on our understanding of brain function (Aso et al., 2009; Baslow et al., 2012). However, at the present time there has been no rigorous validation of the methodology or thorough explanation of the physiological basis for the effects (Miller et al., 2007; Jin and Kim, 2008; Yacoub et al., 2008). In this commentary we discuss the possible explanations for the functional FA observations and how future studies could begin to explore these effects.

The most likely explanation for the observed increase in FA is that it reflects changes in the BOLD fMRI signal. It is well established that neuronal activation is associated with a decrease in the transverse relaxation rate (*R*_2_), observed as an increase in the gray matter (GM) magnetic resonance signal (Ogawa et al., 1990). In contrast, WM BOLD activation is a very rarely reported phenomenon. It follows that the relative GM/WM BOLD signal ratio is very likely to *increase* during a stimulus-induced positive BOLD period, and *decrease* during the post-stimulation negative BOLD period. Since GM and WM have different FA-values, a change in the relative GM/WM ratio may have an impact upon FA quantification. In contrast, since GM and WM have similar MD values, a change in the GM/WM ratio would probably not influence MD. However, the very small BOLD signal changes observed in this study would seem to suggest otherwise, but could be explained by the method of analysis. By taking into account voxels along the entire tract length, areas of WM proximal to GM regions at tract termination points might have been more strongly influenced by a GM BOLD effect than those in the main body of the tract.

To test this hypothesis we simulated the effect which a partial-volume of gray matter would have on parallel and transverse diffusivity using published parameters. Relaxation rates *R*_2__*gm* = 14.12 1/s, *R*_2__*gm*_activation_ = 14.00 1/s, and *R*_2__*wm* = 12.34 1/s; estimated from the relation $\Delta R_{2}=-\frac{\Delta S}{S}/TE$ (Donahue et al., 2006; Miller et al., 2007); *ADC* values *ADC_gm_* = 0.937 * 10^−3^ mm^2^/s, *ADC_wm_*,_parallel_ = 1.5*10^−3^ mm^2^/s, *ADC*_*wm*,radial_ = 0.4*10^−3^ mm^2^/s (Kiselev and Il'yasov, 2007; Qiu et al., 2008); Gray matter fraction (*f_gm_*), White matter fraction (*f_wm_* = 1 − f_gm_), TE (78 ms) and *b*-value (1000 s/mm^2^) (Mandl et al., 2008) using the equation below:

$$\begin{array}{l} \frac{\Delta S}{S}=\left(\frac{S_{activation}}{S_{baseline}}-1\right)*100\%\\ =\left(\text{​}\frac{\begin{array}{c} f_{gm}\cdot e^{-R_{2,gm,act}\cdot TE-ADC_{gm}\cdot bvalue}\\ +f_{wm}\cdot e^{-R_{2,wm}\cdot TE-ADC_{wm{,}_{radial}^{par}}\text{​​​​}\cdot bvalue} \end{array}}{\begin{array}{c} f_{gm}\cdot e^{-R_{2,gm}\cdot TE-ADC_{gm}\cdot bvalue}\\ +f_{wm}\cdot e^{-R_{2,wm}\cdot TE-ADC_{wm{,}_{rad}^{par}}\cdot bvalue} \end{array}}-1\text{​}\right) \end{array}$$

**Figure 1:**
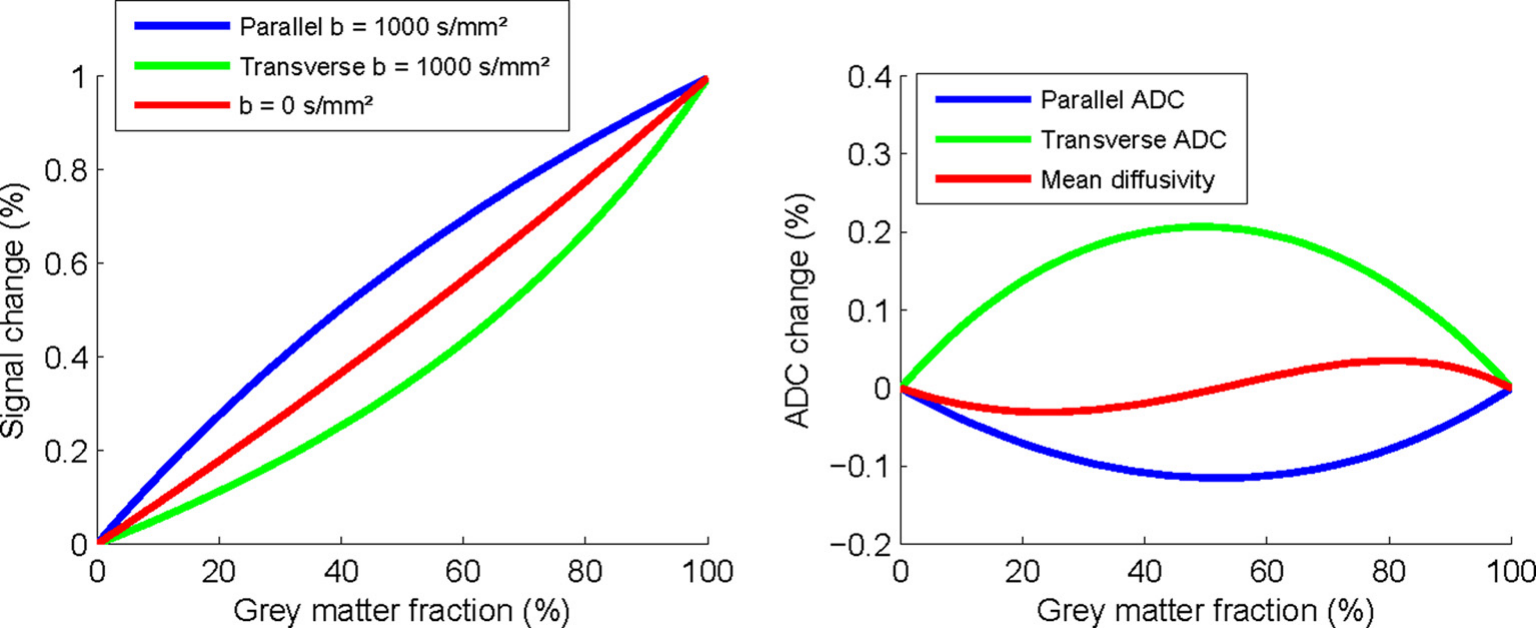
**Simulated changes in parallel and transverse diffusivity signal and ADC as a function of percentage partial-volume with gray matter**.

Figure 1 illustrates that the signal changes are substantial even with modest 20% gray matter partial volumes, with a 0.28% increase in parallel diffusivity, 0.11% reduction in transverse, and BOLD change of 0.18%. This suggests that small BOLD changes could provide a physiological explanation for the changes observed. However, this possibility would still not explain the differences in observed time courses between the two stimulation types. Although changes in the GM BOLD signal would appear to be the most likely explanation, it is still unclear to what extent and precisely how this could impact on FA measurements in central white matter pathways.

A more technical consideration is the possible effect of image noise and partial volumes on FA quantification (Basser and Jones, 2002; Rudrapatna et al., 2012). At 2.5 × 2.5 × 7 mm^3^ resolution, it is likely that several WM voxels could be contaminated with volumes of GM, even after using standardized white matter templates. Noise in MRI acquisitions is thought to cause an overestimation of FA in both isotropic and anisotropic structures (Pierpaoli and Basser, 1996), and it is also well known that stimulation-evoked BOLD responses demonstrate substantial trial-to-trial fluctuations. Therefore, could the trial-to-trial BOLD response fluctuations impose an apparent increase in the MR noise level and cause a functional FA overestimation? Although a possibility, the very low BOLD signal changes indicate that this is unlikely. The specificity of the results to pathways previously associated with tactile or visual function, and the replication of prior results (Mandl et al., 2008) suggest that partial volume or noise effects cannot fully explain these findings.

A final possibility is that FA increases may reflect activity-evoked glial swelling associated with increases in extracellular potassium levels (Ransom et al., 1985). Such activity would predict an increase in Na^+^, K^−^-ATPase utilization to recover post-activation transmembrane ion gradients, which in turn might translate into changes in vascular oxygenation levels. However, the extant evidence from BOLD fMRI and PET studies does not support a metabolic explanation for the observed effects. *In vitro* studies in the rat brain—which are free from confounding vascular effects - show that massive depolarization and increases in metabolism have a minimal effect upon WM ADC quantification (Anderson et al., 1996). Thus, the lack of convincing evidence for WM activation is in line with the emerging view that WM energy consumption is predominantly dedicated to non-signaling related ATP consumption and maintenance of resting potentials (Harris and Attwell, 2012).

In order to advance the use of functional DTI, a more detailed exploration of the origin of the observed changes is vital. To describe the basic WM, GM, and CSF model, even when contributions from blood and *R*_2_ are excluded, requires 18 separate parameters (Basser and Jones, 2002). This level of complexity sets significant limitations on the interpretation of a functional FA change, therefore we recommend caution when interpreting the origin of fDTI signals, as at the present time the picture is far from clear. Future investigations should: (1) exclude activated BOLD voxels from FA analyses to ameliorate the impact of possible BOLD or noise effects and (2) investigate the effect of hypercapnia on FA quantification in humans, since this is not associated with a substantial increase in neuronal information processing. Such experiments may help disentangle the impact of vascular effects upon functional FA quantification and extend our understanding of signal changes in WM using fDTI.

### Conflict of interest statement

The authors declare that the research was conducted in the absence of any commercial or financial relationships that could be construed as a potential conflict of interest.
